# Weakened IL-15 Production and Impaired mTOR Activation Alter Dendritic Epidermal T Cell Homeostasis in Diabetic Mice

**DOI:** 10.1038/s41598-017-05950-5

**Published:** 2017-07-20

**Authors:** Zhongyang Liu, Guangping Liang, Li Gui, Yashu Li, Meixi Liu, Yang bai, Xiaorong Zhang, Xiaohong Hu, Jian Chen, Chibing Huang, Xusheng Liu, Gaoxing Luo, Jun Wu, Weifeng He

**Affiliations:** 1 State Key Laboratory of Trauma, Burn and Combined Injury, Institute of Burn Research, Southwest Hospital, The Third Military Medical University, Chongqing, 400038 China; 2 Department of Plastic Surgery, The First Affiliated Hospital of Zhengzhou University, Zhengzhou, 450000 Henan P.R. China; 3 Chongqing Key Laboratory for Disease Proteomics, Chongqing, 400038 China; 4 Department of Neurology, Southwest Hospital, Third Military Medical University, Chongqing, 400038 China; 5 Department of Burns, The First Affiliated Hospital of Sun Yat-sen University, Guangzhou, 510080 Guangdong P.R. China; 6 Department of Urology, Xinqiao Hospital, The Third Military Medical University, Chongqing, China

## Abstract

Diabetes is associated with impaired wound healing, which may be caused primarily by a deficiency in dendritic epidermal T cells (DETCs). In the epidermis, IL-15, IGF-1, and mTOR are known to regulate the maintenance of DETCs; however, it is unclear how these molecules may intersect to regulate DETC homeostasis in diabetes. Here, we show that the reduction of DETCs in the epidermis of diabetic mice is caused by altered homeostasis mediated by a reduction in IL-15 levels. Both impaired mTOR activation and reduction of IL-15 in the epidermis play important roles in DETC homeostasis. Moreover, IGF-1 drives keratinocytes to produce IL-15. The activation of IL-15 is dependent on mTOR, and conversely, mTOR regulates IGF-1 production in DETC, in a classic feedback regulatory loop. Our data suggest that in the setting of diabetes, reduced IGF-1, impaired mTOR pathway activation and reduced IL-15 in the epidermis function coordinately to promote altered DETC homeostasis and delayed skin wound closure.

## Introduction

Diabetes is associated with impaired wound healing^1, 2^, which may be due in part to a deficiency of IGF-1 in diabetic skin^3^. Dendritic Epidermal T Cells (DETCs) contribute to wound repair by producing IGF-1 and KGF^4–6^. Large numbers of activated DETCs at the wound margin are required for IGF-1/KGF production in the epidermis for efficient wound healing. Recently, altered homeostasis of DETCs has been demonstrated in epidermis of type II diabetes^7^. However, the precise mechanisms for abnormal homeostasis of DETCs in type I diabetes remain to be clarified.

Both TCR signaling and growth cytokines are essential for T cell maintenance. Several secreted factors have been shown to contribute to the development and activation of DETCs upon TCR engagement in non-diabetic mice. IL-15, which is similar to IL-2 in its biological properties and three-dimensional configuration, is the most important growth factor in the epidermis for T cell survival and proliferation upon TCR engagement, given that IL-15, rather than IL-2, is expressed in the epidermis under physiological conditions. Consistently, previous studies have clearly demonstrated that epidermal IL-15 is required for DETC development and homeostasis in the epidermis^8, 9^. However, DETC homeostasis is normal both in IRF-1 (−/−) mice (9), which exhibit reduced epidermal IL-15 levels, and in IL-15 transgenic mice^10^, which overexpress IL-15. These observations suggest that alterations in epidermal IL-15 levels within a specific tissue are unlikely to affect DETC homeostasis, whereas over threshold amounts of IL-15 potentially alter the development and homeostasis of DETCs in the epidermis. IGF-1, which is secreted exclusively by DETCs in the epidermis, enhances the activation of DETCs to further produce IGF-1 upon TCR stimulation *in vitro* ^6^ and potentially is involved in regulating DETC maintenance. Furthermore, inhibition of the Akt/mTOR pathway, a central regulator of metabolism in the pathogenesis of diabetes^11–13^, is suggested to have a negative impact on DETC homeostasis, potentially through its inhibition of TCR expression on DETCs^14^. These results suggest that IL-15, IGF-1, and mTOR pathways each can regulate DETC homeostasis. However, whether modulation of these pathways is responsible for the altered DETC homeostasis in diabetic mice and what internal connections exist among them in the context of diabetes remain to be answered.

In the present study, we showed that weakened IL-15 production contributes to the altered DETC homeostasis in type I diabetes induced by STZ. Furthermore, keratinocyte-derived IL-15, as one of major sources of IL-15 in the epidermis, is regulated by IGF-1 via an mTOR-dependent pathway. These results reveal a mechanism by which the IL-15, IGF-1 and mTOR pathways interconnect to impede DETC homeostasis in diabetic mice, which may explain the impaired wound healing that occurs in diabetes.

## Results

### Altered homeostasis of DETCs contributes to abnormal wound healing in diabetic mice

**Figure 1:**
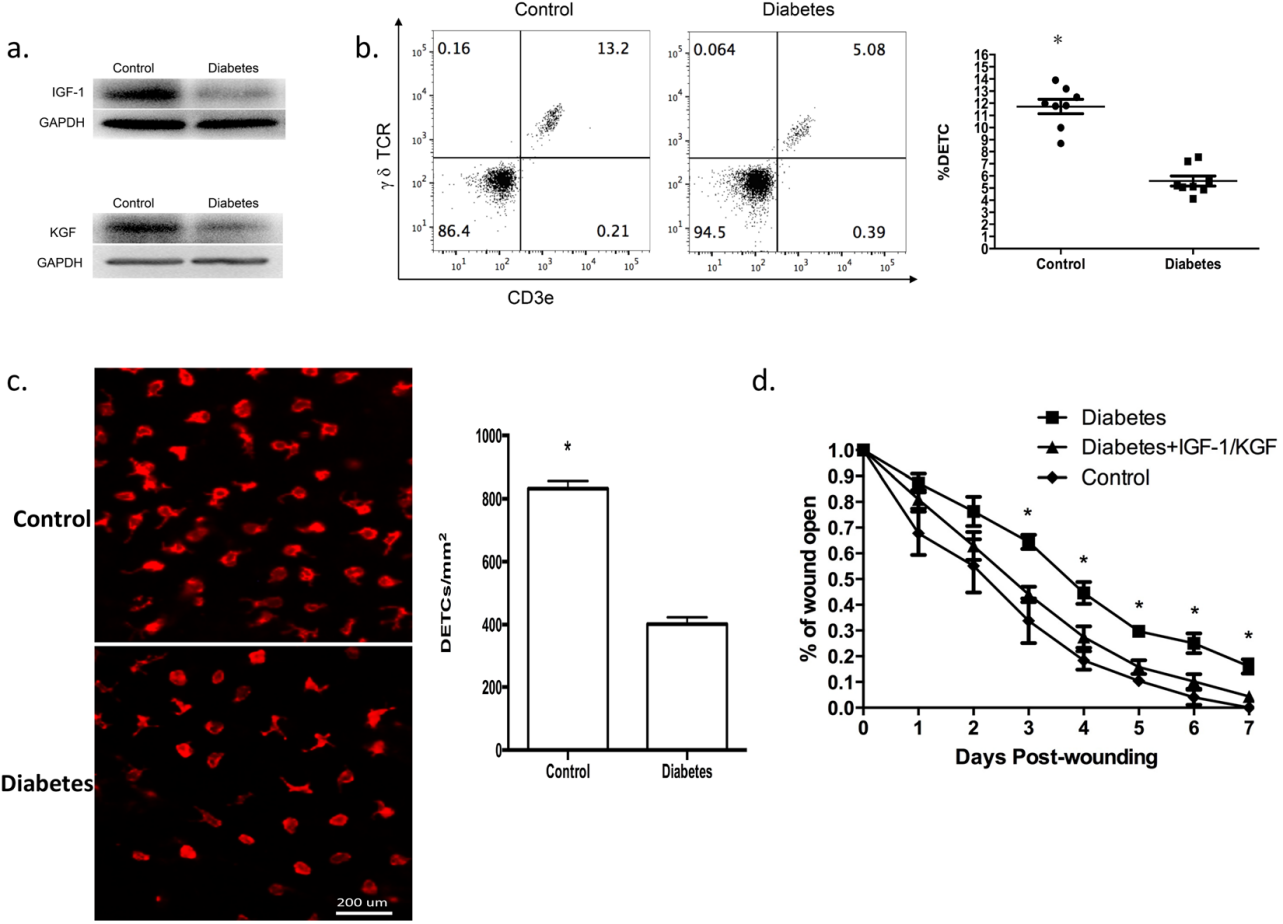
The reduced numbers of DETCs in diabetic mice may affect wound healing by reducing IGF-1/KGF-1 production. (**a**) 6–8 weeks wild-type male C57BL/6J mice were administered STZ to induce diabetes or vehicle control daily for 6 days. The mice were administered full-thickness wounds in their back skin 4 weeks later. At day 1 after wounding, epidermal sheets around wounds in STZ-induced diabetic or control mice were excised to assess IGF-1 and KGF production by Western analysis. (**b**) Single cell suspensions of epidermal sheets around wounds were stained with γ δ TCR and CD3e antibodies and analyzed by FACS. (**c**) TCR γδ immunofluorescence staining of epidermal sheets from STZ-induced diabetic or control mice, the number of γ δ T cells was quantified per mm^2^. (**d**) Application of IGF-1 and KGF to the wounds promoted wound healing in STZ-induced diabetic mice. All data represent at least three independent experiments and each experiment includes 2 animals (one control and one experimental mouse). All error bars represent mean ± SEM. *P < 0.05.

DETCs contribute to efficient wound healing by secreting IGF-1 and KGF-1^5, 6^. To investigate whether decreased IGF-1/KGF-1 secretion by DETCs causes delayed wound healing in diabetic mice, C57 mice were subject to 6 daily treatments with vehicle control or with streptozotocin (STZ) to induce diabetes^15^. Four weeks later, the mice were administered full-thickness wounds in their back skin^16^. Compared with wild-type controls, diabetic mice produced less IGF-1/KGF (Fig. 1a) and had substantially fewer DETCs (Fig. 1b and c) in the epidermis around their wounds. However, IGF-1/KGF-1 administration restored wound healing in the diabetic mice nearly to the control level (Fig. 1d). Because IGF-1/KGF-1 in the epidermis is primarily produced by DETCs^5, 14^, these results suggest that the reduced numbers of DETCs in diabetic mice may affect wound healing by leading to reduction of IGF-1/KGF-1.

**Figure 2:**
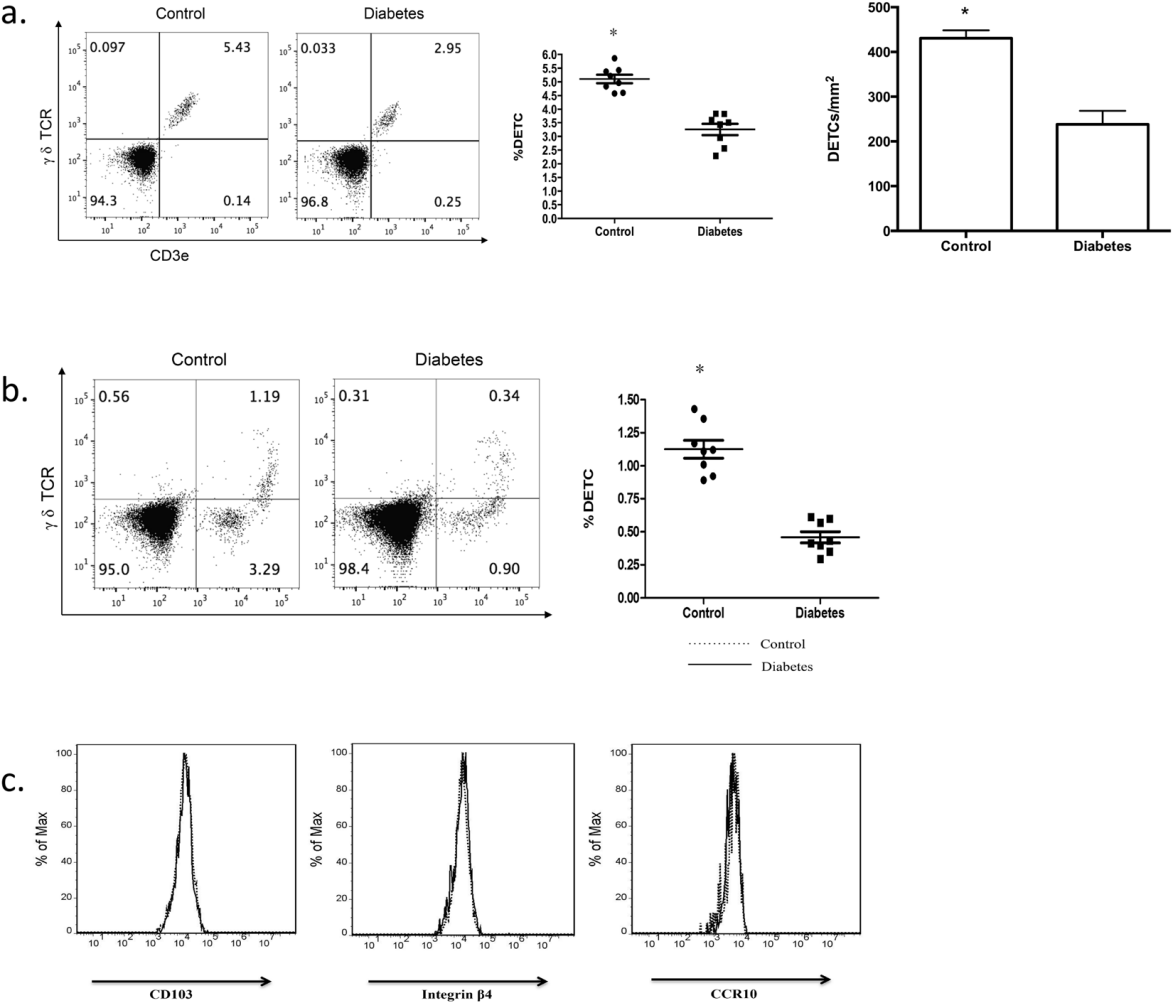
The reduction of DETCs in diabetic mice is likely to result from altered homeostasis without enhanced egression. 6–8 weeks wild-type male C57BL/6J mice were administered STZ to induce diabetes or vehicle control daily for 6 days. The mice were administered full-thickness wounds in their back skin 4 weeks later. At day 1 after wounding, epidermal sheets around wounds in STZ-induced diabetic or control mice were excised. (**a**) Single cell suspensions of epidermal sheets were stained with γ δ TCR and Vγ3 antibodies. (**b**) Single cell suspensions of dermal sheets from control and diabetic mice without wounds were stained with γ δ TCR and Vγ3 antibodies. (**c**) The expression levels of CD103, integrin β4, and CCR10 on DETCs from STZ-induced diabetic or control mice were examined by means of FACS. All data represent at least three independent experiments and each experiment includes 2 animals (one control and one experimental mouse). All error bars represent mean ± SEM. * *P* < 0.05.

To further evaluate the cause for reduced numbers of DETCs in diabetic mice, we assessed whether DETC levels were also reduced in intact skin of diabetic mice. Our results confirmed that diabetic mice exhibited notable DETC reduction in intact skin (all of epidermal γδ T cells are Vγ3 positive, Fig. 2a). Because Vγ3/δ1T cells (DETC precursors) are generated exclusively during a short period in the fetal thymus before they develop in the epidermis^17, 18^, DETC reduction in intact skin could potentially be explained by DETC egression from the epidermis and/or altered DETC homeostasis in diabetic mice. In fact, altered DETC homeostasis in diabetic mice has been reported^7^, but the role of DETC egression in diabetic mice has not been evaluated. Thus, to determine whether DETC egression contributes to the DETC reduction in diabetic mice, we examined the number of DETCs in the dermis and peripheral lymph node. The dermis of diabetic mice had fewer DETCs than the dermis of control mice (Fig. 2b), while lymph node DETCs were not detected in either diabetic or control mice (data not shown). Additionally, DETCs from diabetic and control mice expressed comparable levels of αE, integrin β4 and CCR10 as those from control mice (Fig. 2c). These adhesion molecules and chemokine receptors are crucial for DETCs residence in the epidermis^19, 20^. Therefore, the reduced number of DETCs in diabetic mice is likely to result from altered homeostasis without enhanced egression.

### Impaired mTOR pathway and weakened IL-15 production correlate with reduced DETC homeostasis in diabetic mice

**Figure 3:**
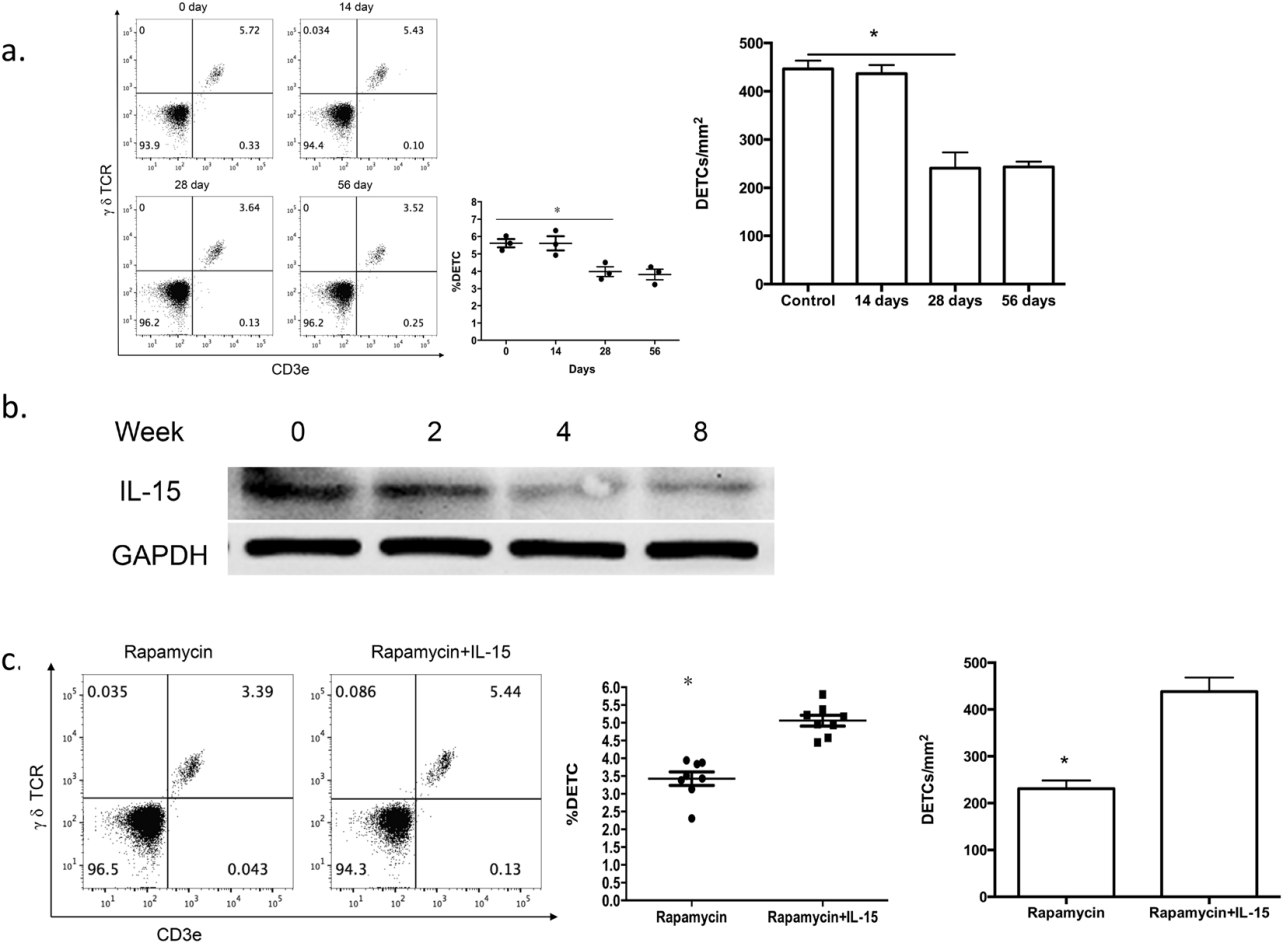
Addition of IL-15 recovers rapamycin-mediated abnormal DETC homeostasis. (**a,b**) 6–8 weeks wild-type male C57BL/6J mice were administered rapamycin or vehicle control daily for 8 weeks. At 8 weeks after administration of the vehicle control and at, 2, 4, and 8 weeks after rapamycin treatment, epidermal sheets were obtained for assessing the number of DETCs by FACS (panel a) and the production of IL-15 by Western analysis (panel b). (**c**) IL-15 (1 μg) or vehicle control was intradermally injected daily on the back skin of rapamycin-treated mice for 3 days, and epidermal sheets were excised for assessing the number of DETCs by FACS. All data represent at least three independent experiments and each experiment includes 6 animals (3 control and 3 experimental mice). All error bars represent mean ± SEM. * *P* < 0.05.

The Akt/mTOR pathway controls metabolism and plays a pivotal role in the pathogenesis of diabetes^11–13^. Weakened mTOR pathway activation has been observed in the intact skin of STZ-induced diabetic rats^21^. Furthermore, mTOR pathway inhibition is suggested to inhibit DETC homeostasis by inducing autophagy and blocking proliferation^14^. To verify the role of the mTOR pathway in mediating DETC homeostasis, we treated mice with the mTOR inhibitor rapamycin for 2, 4, or 8 weeks. Consistent with previous results^14^, 2 weeks of sustained rapamycin treatment failed to reduce the number of DETCs in wild-type C57 mice; however, 4 or 8 weeks of rapamycin treatment led to a marked reduction in the number of DETCs (Fig. 3a). These results suggest that the impairment of the mTOR pathway alters DETC maintenance.

To identify additional pathways that may mediate the response to mTOR inhibition, we examined additional signaling pathways known to regulate DETC homeostasis. Given the critical role of IL-15 in DETC development and maintenance in the epidermis^8, 9^, we hypothesized that IL-15 may contribute to the rapamycin-mediated reduction of DETCs. Indeed, the production of IL-15 in the epidermis was gradually reduced, with substantially lower levels at 4 and 8 weeks after rapamycin treatment, which mirrors the pattern of DETC reduction after rapamycin treatment (Fig. 3b). Additionally, intracutaneous injection of IL-15 restored the number of local DETCs in rapamycin-treated mice (Fig. 3c), providing direct evidence that IL-15 mediates the effects of rapamycin on DETC homeostasis.

**Figure 4:**
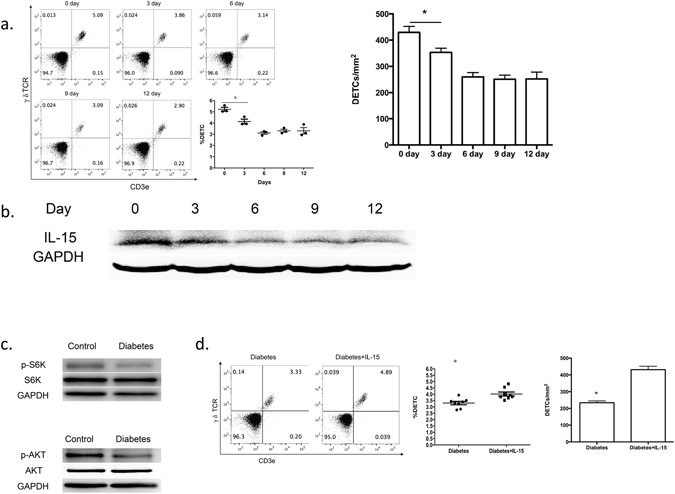
Impaired mTOR pathway and weakened IL-15 production correlate with altered DETC homeostasis in diabetic mice. (**a,b**) 6–8 weeks wild-type male C57BL/6J mice were administered STZ for 6 days. At day 0, 3, 6, 9, and 12 after STZ treatment, epidermal sheets were excised for assessing the number of DETCs by FACS (panel a) and the production of IL-15 by Western blotting (panel b). (**c**) The phosphorylated and total expression of S6K and Akt in the epidermis of diabetic mice and wild-type controls was assessed by means of Western blotting. (**d**) IL-15 (5 μg) or vehicle control was intradermally injected daily on the back skin of STZ-treated mice for 3 days, and epidermal sheets were excised for assessing the number of DETCs by FACS. All data represent at least three independent experiments and each experiment includes 6 animals (3 control and 3 experimental mice). All error bars represent mean ± SEM. * *P* < 0.05.

To determine whether a similar pathway of mTOR and IL-15 suppression may explain the reduced DETC homeostasis in the STZ diabetes model, we examined the effects of diabetes induction over a timecourse of STZ treatment. The number of DETCs (Fig. 4a) and the production of IL-15 in the epidermis (Fig. 4b and Figure S1) both followed patterns of reduction by day 3 after drug treatment and remained low until day 12. Furthermore, similar to previous findings in rats^21^, the phosphorylation of the mTOR pathway proteins S6K and Akt was suppressed in the epidermis of STZ-treated mice (Fig. 4c). Additionally, intracutaneous injection of IL-15 mediated a small but significant increase in the number of local DETCs in STZ-treated mice (Fig. 4d), perhaps due to the severely impaired TCR expression and mTOR signaling pathway in DETCs. These data further support the possibility that IL-15 functions downstream of mTOR to regulate DETC homeostasis and suggest that the reduced mTOR activation and IL-15 production in the epidermis of diabetic mice may explain their dysfunctional DETC maintenance.

### mTOR pathway is required for regulation of keratinocyte-derived IL-15 by IGF-1 in vitro

**Figure 5:**
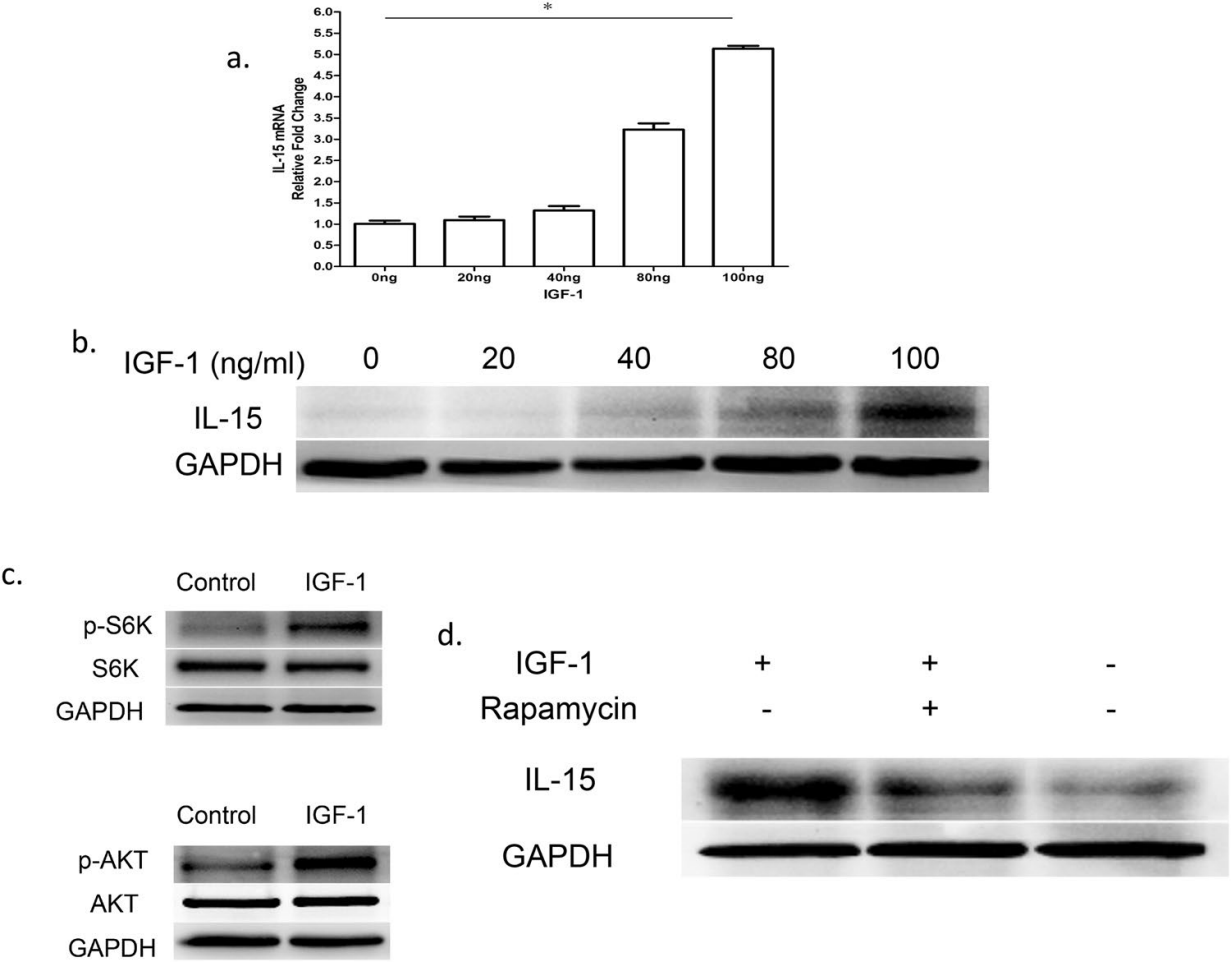
IGF-1 regulates keratinocyte-derived IL-15 in an mTOR-dependent manner. (**a,b**) Primary keratinocytes were isolated from newborn C57BL/6J male wild-type mice, and CD11c^+^ cells were depleted by MACS. These cells were cultured for 1 day in 0, 20, 40, 80, or 100 ng/ml IGF-1. IL-15 was assessed at the mRNA level by real-time PCR (panel a) and at the protein level by Western blotting (panel b). (**c**) Phosphorylated and total S6K, and Akt in keratinocytes were measured in the presence or absence of IGF-1 (100 ng/ml, for 24 hours) by Western blotting. (**d**) Analysis of IGF-1-induced IL-15 production suppression by rapamycin in keratinocytes after 24 h culture. All data represent at least three independent experiments. All error bars represent mean ± SEM. * *P* < 0.05.

Keratinocytes are a major source of IL-15 in the epidermis^22^. Furthermore, keratinocytes can respond to IGF-1 via IGFR for their survival and proliferation^23^. Therefore, we hypothesized that IGF-1 may regulate IL-15 production in keratinocytes. To address this possibility, primary keratinocytes (CD11C+ cells depleted by MACS) were isolated from newborn C57 wild-type mice^24^ and cultured for three days in the presence of various doses of IGF-1 (0–100 ng/ml). Our results demonstrate that IGF-1 promotes IL-15 production by keratinocytes in a dose-dependent manner at both the mRNA (Fig. 5a) and protein levels (Fig. 5b). These findings suggest that IL-15 produced by keratinocytes regulates IGF-1 production.

To further investigate whether the mTOR pathway is a mediator of IGF-1-dependent IL-15 production, we examined the effect of IGF-1 on mTOR pathway activation. IGF-1 activated S6K and AKT in keratinocytes (Fig. 5c), which confirms that IGF-1 functions upstream of mTOR. To determine whether IGF-1-mediated IL-15 production in keratinocytes is dependent on mTOR signaling activation, we stimulated primary keratinocytes with IGF-1 in the presence of rapamycin. Rapamycin markedly attenuated the increase in IL-15 production by IGF-1 (Fig. 5d). Taken together, these results suggest that IGF-1 stimulates keratinocytes to produce IL-15 via the mTOR pathway.

### IGF-1 regulates epidermal IL-15 production in mTOR-dependent manner *in vivo*

**Figure 6:**
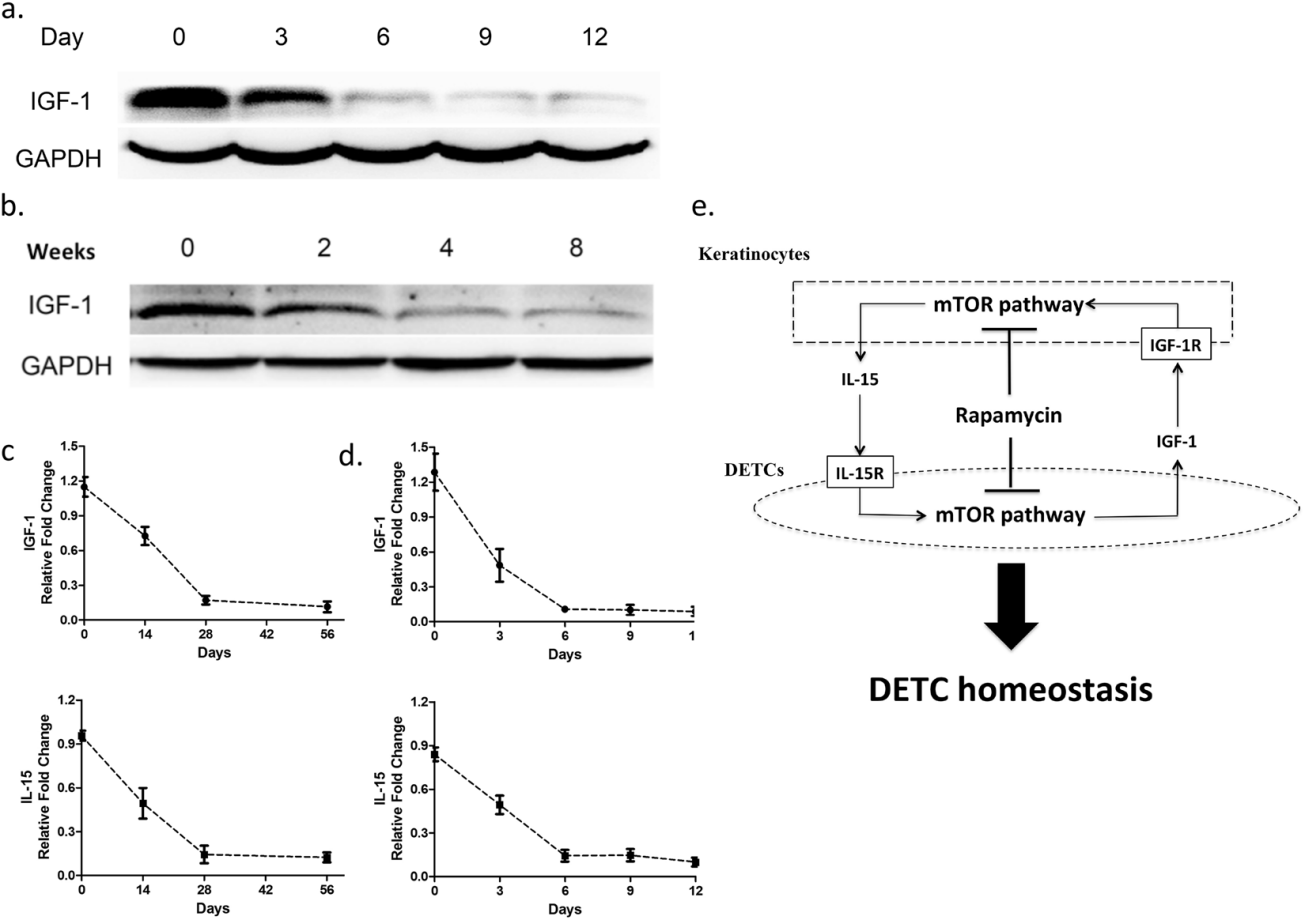
Both productions of IGF-1 and IL-15 in epidermis are regulated by mTOR pathway. (**a**) 6–8 weeks wild-type male C57BL/6 J mice were administered STZ daily for 6 days. At day 0 (no treatment), 3, 6, 9, and 12 after STZ treatment, epidermal sheets were obtained for detecting the production of IGF-1 by Western blotting. (**b**) Wild-type C57BL/6J mice were administered rapamycin daily for 8 weeks. At 0, 2, 4, and 8 weeks after rapamycin treatment, epidermal sheets were obtained for detecting the production of IGF-1 by Western blotting. (**c**) The kinetics of IL-15 and IGF-1 in rapamycin-treated mice is shown. (**d**) The kinetics of IL-15 and IGF-1 in STZ-treated mice is shown. All data represent at least three independent experiments and each experiment includes 6 animals (3 control and 3 experimental mice). All error bars represent mean ± SEM. * *P* < 0.05. (**e**) Model of signaling network that regulates wound healing during diabetes.

While our study supports a role for mTOR activation downstream of IGF-1, recent research also suggests that conversely, mTOR can induce diabetic symptoms in a percentage of patients and suppress IGF expression and signaling in skeletal muscle^25^. Therefore, we speculated that mTOR might regulate IGF-1 expression in a feedback loop in STZ-induced mice. To determine whether the mTOR pathway regulates IGF-1 production, we assessed the effects of rapamycin on IGF-1 expression *in vivo*. Our results demonstrate that STZ (Fig. 6a) or rapamycin (Fig. 6b) treatment of mice reduces IGF-1 expression as assessed by Western blotting of epidermal sheets, which include keratinocytes, Langerhans, and DETCs. Furthermore, IL-15 and IGF-1 in the epidermis were regulated by rapamycin (Fig. 6c) and STZ (Fig. 6d) under similar kinetics, which provides additional support for the coordinated suppression of IGF-1 and IL-15 in diabetes.

## Discussion

IGF-1, which is exclusively secreted by DETCs in the epidermis^14^, plays a critical role in keratinocyte maintenance^6^. In diabetes, low levels of IGF-1 and consequent marked DETC reduction in the epidermis contributes to the pathophysiological characteristics of diabetic skin, which negatively impacts wound healing^3, 26^. Inhibition of IGF-1 production in DETCs *in vitro* and *in vivo* upon administration of the mTOR inhibitor rapamycin reduces TCR stimulation of DETCs^14^, which is required for DETC survival, activation, and proliferation^27, 28^. Additionally, IL-15 is required for DETC development and homeostasis in the epidermis^8, 9^. However, over-expression of IL-15 in transgenic mice unsuccessfully alters DETC homeostasis^10^, which suggests that a threshold level of IL-15 mediates its effects on DETCs. Thus, IGF-1, mTOR and IL-15 all are suggested to regulate homeostasis of DETCs, but the coordinate regulation of these pathways of DETC homeostasis in diabetes is not well characterized.

To further understand the process of homeostasis of DETCs, including the coordinated roles of IGF, mTOR and IL-15, we examined homeostasis in the STZ mouse model of diabetes. Our results verify that diabetes induction disrupts DETC homeostasis, which can be observed at the site of scarring and in intact epidermis and can be improved by administration of IL-15 after STZ treatment. The decrease in the numbers of DETCs upon diabetes induction is most likely explained by homeostasis, rather than egression, given that diabetic and control mice expressed comparable levels of αE, integrin β4 and CCR10.

Given the well-established link between mTOR signaling and diabetes^11–13^, we also examined effects of mTOR pathway inhibition on IL-15 production. Our results demonstrate that IL-15 is suppressed coordinately with DETC homeostasis upon treatment with rapamycin in wild-type mice. Furthermore, effects of rapamycin on DETC homeostasis are reversed by the co-administration of wild-type mice with IL-15, which suggests that mTOR functions upstream of IL-15 and that IL-15 may comprise a key factor in the mTOR pathway toward DETC homeostasis. Suppression of IL-15 and suppressed phosphorylation of several components of the mTOR pathway, including S6K and AKT, occur upon treatment of mice with STZ, which further supports their coordinate role in diabetes.

Using purified keratinocytes, we also demonstrated that IGF-1 stimulates keratinocytes to produce IL-15 and activates mTOR signaling. The activation of IL-15 is inhibited by rapamycin, which suggest that the effects of IGF-1 on IL-15 are mTOR-dependent. Furthermore, mTOR inhibition suppresses IGF-1, and the production of IGF-1 and IL-15 in the epidermis exhibit similar kinetics in STZ-induced diabetic mice and wild-type animals with rapamycin treatment. Taken together, these results support a pathway in which reduced IGF-1 in diabetes leads to suppressed activation of the mTOR pathway, which further reduces IGF-1. The reduction in mTOR activation also leads to attenuated production of IL-15, which suppresses DETC homeostasis. Suppressed DETC numbers, in turn, leads to reduced production of IGF and KGF, which further delays wound healing (Fig. 6e).

Although our results suggest that IL-15 production by keratinocytes is a key mediator of DETC homeostasis, Langerhans cells (LCs) provide another important source of IL-15 in the epidermis^29^. LC-derived IL-15 is regulated in an IGF-1-independent manner, as there is no evidence that IGF-1 acts directly on LCs. How LC-derived IL-15 is regulated and whether it contributes to abnormal DETC homeostasis in diabetic mice needs further clarification. Furthermore, although mTOR pathway was shown here as key regulator for IL-15 production by keratinocytes, rapamycin failed to eliminate IL-15 production by keratinocytes totally *in vitro* and *in vivo*. It suggested that other signaling was deeply involved in regulation of IL-15 expression by keratinocytes in mTOR-independent manner. In addition, NKG2D and Skint1 have also been mentioned as “first signaling” molecules that directly activate DETCs^30, 31^, and the roles of these molecules in the altered DETC homeostasis of diabetic mice warrnt further study.

## Materials and Methods

### Animals

C57BL/6 WT (H-2b) mice were purchased from the Experimental Animal Center in the Third Military Medical University in Chongqing, China. All mice were housed under specific pathogen-free conditions at the Southwest Hospital Experimental Center (Chongqing, China), and given food and water *ad libitum*. Male mice aged 6–8 weeks were used for all experiments. All experimental methods described above were conducted in accordance with guidelines for animal care and were approved by the First Affiliated Hospital (Southwest Hospital) of the Third Military Medical University. All experiments were approved by the Laboratory Animal Welfare and Ethics Committee of the Third Military Medical University.

### Streptozotocin (STZ)-induced diabetic animal model

Mice were injected i.p. with 150 μl of STZ (100 mg/kg, Sigma-Aldrich, USA) or the vehicle control for 6 consecutive days. Venous blood glucose levels were measured in non-fasting animals using a glucometer. Mice were evaluated every 2 days at 2:00 p.m. and were considered diabetic when the blood glucose levels were sustained above 250 mg/dL. STZ has not been reported to directly affect activation of mTOR signaling, production of IL-15 or IGF in epidermal cells, including keratinocytes and DETCs.

### Wounding procedure

Wounding was performed on mice anesthetized with sodium pentobarbital. Briefly, the dorsal surface of the mouse was shaved, the back skin and panniculus carnosus were pulled up, and one or two sets of sterile full-thickness wounds were generated using a sterile 4-mm punch tool. In some experiments, 100 ng recombinant IGF-1 and KGF (R&D Systems, USA) or control buffer alone was applied to each wound site immediately post-wounding and daily thereafter. For rapamycin administration, mice were injected i.p. daily with 200 μl of vehicle control or 1% rapamycin (Selleck Chemicals, Houston) in 0.2% carboxymethyl cellulose and 0.25% Tween 80 (Sigma-Aldrich, USA) in distilled H_2_O.

### Isolation of epidermal sheets

The skin harvested from STZ-induced diabetic and control mice was washed twice in sterile PBS. Next, the skin was cut into 5 mm × 5 mm pieces and washed again with PBS. The pieces were digested with 0.5 g/l Dispase II (Sigma, USA) at 37 °C for 1–2 hours, and then the epidermis and dermis were separated carefully. The epidermal sheet was minced and digested with 0.5% trypsin at 37 °C for 10 minutes, followed by cell collection via centrifugation. The cells were suspended in RPMI 1640 Medium (GIBCO BRL, Gaithersburg, MD) supplemented with 10% fetal bovine serum, 100 mg/ml of streptomycin, 100 U/mL penicillin and 2 mM glutamine (Hyclone, USA).

### Primary keratinocyte isolation and culture

Primary keratinocytes were isolated from newborn C57 mice as previously described^24^. The isolated cells were re-suspended in Serum-Free Keratinocyte Medium (K-SFM, GIBCO, 17005) with human recombinant epidermal growth factor (0.1–0.2 ng/ml), bovine pituitary extract (20–30 mg/ml), mouse epidermal growth factor (10 ng/ml; BD, 354001), cholera toxin (1 × 10^−10^ M; Sigma, C9903), calcium chloride (0.05 mM) and penicillin and streptomycin solution (100 IU/ml, GIBCO, 15140122). The cells were counted and cultured under 5% CO_2_ at 37 °C in an incubator. Culture medium was replenished every 2–3 days. In some experiments, primary keratinocytes (CD11c^+^ cells depleted by MACS) were cultured for one day in various doses of IGF-1 (0, 20, 40, 80, and 100 ng/ml). In other cases, primary keratinocytes were stimulated with IGF-1 (100 ng/ml) in the presence of rapamycin (50 nM, Selleck Chemicals, Houston, TX).

### Antibodies and flow cytometry

PerCP CY5.5-conjugated mAb specific for γ δ TCR (GL3 Tianjin Sungene Biotech Co. Ltd) and BV605-conjugated mAbs specific for CD3e (BD Biosciences, USA) were used for flow cytometry. Data acquisition was performed on an Attune Acoustic Focusing Cytometer (Applied Biosystems, Life Technologies, CA, USA), and the data were analyzed using FlowJo software (Tree Star Incorporation, USA). Experiments were repeated at least three times using the same conditions and settings.

### Immunofluorescence

Wounded skins from diabetic or vehicle-treated mice were excised, peeled into halves, and digested in Dispase II. Epidermal sheets were peeled and stained with PE-conjugated anti-γ δ TCR(Tianjin Sungene Biotech Co. Ltd). The images were captured and photographed using Leica fluorescent microscope (CTR6000, Leica, Germany).

### Western blot analysis

Proteins were extracted from cells or epidermal tissue of mice using lysis kits (KeyGEN BioTECH, CA) that were supplemented with 1% protease inhibitor cocktail, 5% phenymethylsulphonyl fluoride and 5% phosphatase inhibitor cocktail according to the manufacturer’s protocol. The lysed cells were scraped, collected and agitated for 20 minutes followed by centrifugation at 14,000 × g for 15 minutes at 4 °C. The supernatant was collected as whole cell lysate, and protein concentrations were determined by BCA protein assays (Thermo Scientific, Rockford, USA). Equal amounts of protein (20 μg) from each sample were loaded onto 10% SDS-PAGE gels for electrophoresis. The separated proteins were transferred to polyvinylidene difluoride (PVDF) membranes (Millipore Immobilon, USA). The membranes were blocked with Tris-buffered saline (TBS) containing 3% bovine serum albumin (BIOSHARP, CA) for 2 hours at room temperature and then incubated with rabbit antibody against IGF-1, KGF (1:200, Santa Cruz Biotechnology, USA); p-S6K (Thr^389^), S6K, p-AKT (Ser^473^), AKT, p-Erk1/2 (Thr^202^/Tyr^204^) (1:1,000, Cell Signaling Technologies, Beverly, MA); IL-15 (1:200, Santa Cruz Biotechnology, USA); or mouse antibody to GAPDH (1:5000, KANGCHEN BIO-TECH, CA) at 4 °C overnight. The membranes were subsequently washed 5 times with TBS containing 0.1% Tween 20 and then incubated with HRP-labeled goat anti-rabbit/mouse secondary antibody (1:5000, ZSGB-BIO, CA) for 1 hour at room temperature. Finally, the membranes were washed 5 times with TBS containing 0.1% Tween 20 and visualized using enhanced chemiluminescence (Pierce, USA) according to the manufacturer’s instructions. The bound antibodies were detected using the ChemiDoc^TM^ XRS Western blot detection system (Bio-Rad, USA).

### Quantitative real-time RT-PCR

Cultured cells were washed with PBS, and total RNA was extracted with an RNeasy® Mini kit (QIAGEN, GER) according to the manufacturer’s instructions. Total RNA was extracted from the epidermal sheets of mouse back skin around a wound using the RNeasy extraction kit (QIAGEN) according to the manufacturer’s instructions. The RNA concentration and quality were measured using a DU800 UV/Vis spectrophotometer (Beckman Coulter, USA), and mRNA was reverse transcribed using First Strand cDNA Synthesis kits (TOYOBO, JPN). Real-time PCR was performed using SYBR Green PCR Master Mix (TOYOBO, JPN) under the following conditions: 95 °C for 2 minutes followed by 50 cycles of 95 °C for 15 seconds, 60 °C for 15 seconds, and 72 °C for 32 seconds. The primers used in this study were as follows:

IL-15:

Forward primer: 5ʹ-GGATTTACCGTGGCTTTGAGTAATGAG-3ʹ

Reverse primer: 5ʹ-GAATCAATTGCAATCAAGAAGTG-3ʹ

GAPDH:

Forward primer: 5ʹ-CGTGCCGCCTGGAGAAAC-3ʹ

Reverse primer: 5ʹ-AGTGGGAGTTGCTGTTGAAGTC-3ʹ

mRNA levels were quantified using the ABI PRISM 7500 Sequence Detection System (Applied Biosystems, Foster City, CA) according to the manufacturer’s protocols. Data were analyzed by the 2-ΔΔ threshold (Ct) method, and GAPDH served as an internal control.

### Statistical analysis

Statistical comparisons were performed using the Student’s *t*-test. Data are presented as the mean ± standard deviation (SD). In all cases, a P value less than 0.05 was considered to be statistically significant.

## Electronic supplementary material

Dataset 1
