# Supplementary material for: Weakened IL-15 Production and Impaired mTOR Activation Alter Dendritic Epidermal T Cell Homeostasis in Diabetic Mice
Source: Sci Rep. 2017 Jul 20;7:6028. doi: 10.1038/s41598-017-05950-5 (PMC5519720; doi:10.1038/s41598-017-05950-5)

Supplementary Legends

Figure S1. The IL-15 production by keratinocytes was impaired in diabetic mice

6-8 weeks wild-type male C57BL/6J mice were administered STZ or vehicle for 6 days. At day 12 after STZ treatment, epidermis tissues were collected and primary keratinocytes were isolated. The isolated keratinocytes cultured with GolgiPlug for 6 hours, then IL-15 protein levels in keratinocytes were detected by means of FACS. Primary rabbit antibody to IL-15 (Santa Cruz Biotechnology, USA) and secondary FITC-conjugated anti-rabbit antibody (ZSGB-BIO, CHINA) was used for intracellular IL-15 staining. All data represent at least three independent experiments. The values were calculated as the mean± SD, *p≤ 0.05.

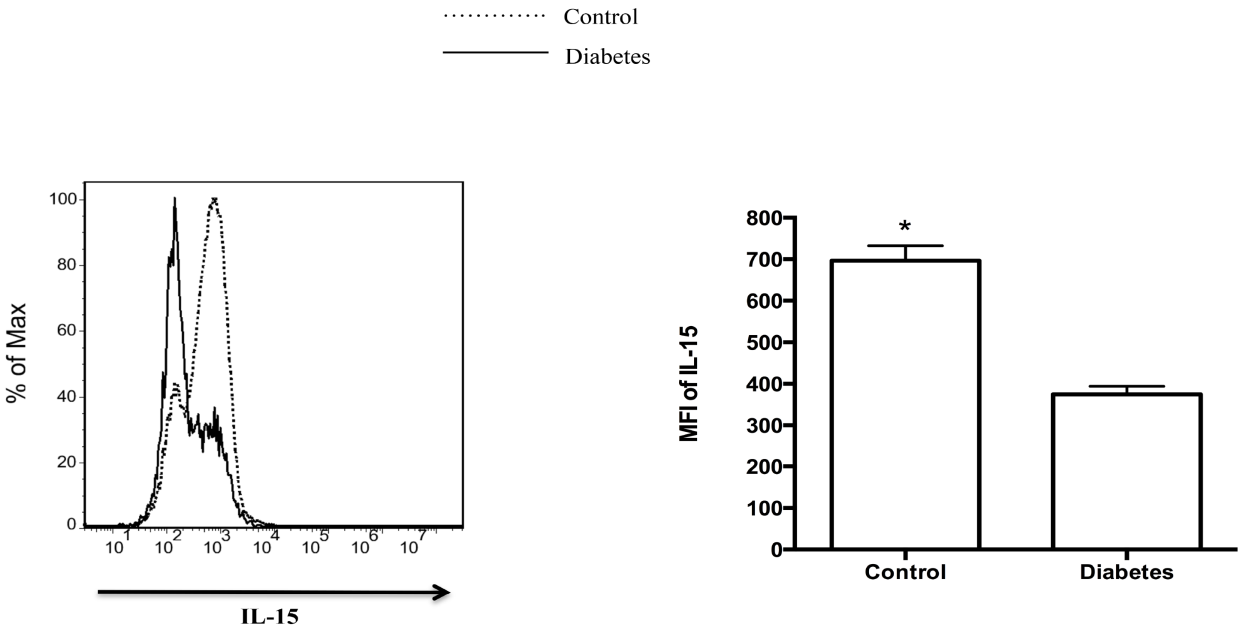

Figure S1

Weakened IL-15 Production and Impaired mTOR Activation Alter Dendritic Epidermal T Cell Homeostasis in Diabetic Mice

Zhongyang Liu[*,#], Guangping Liang[†,#], Li Gui [‡,#], Yang bai[†], Yashu Li[†], Meixi Liu[†], Xiaorong Zhang[†,§], Xiaohong Hu[†,§], Jian Chen[†,§], Chibing Huang[¶], Xusheng Liu[*], Gaoxing Luo[†,§, ##], Jun Wu[†,§, ##], Weifeng He[†,§, ##]

Raw data for Figure 1a

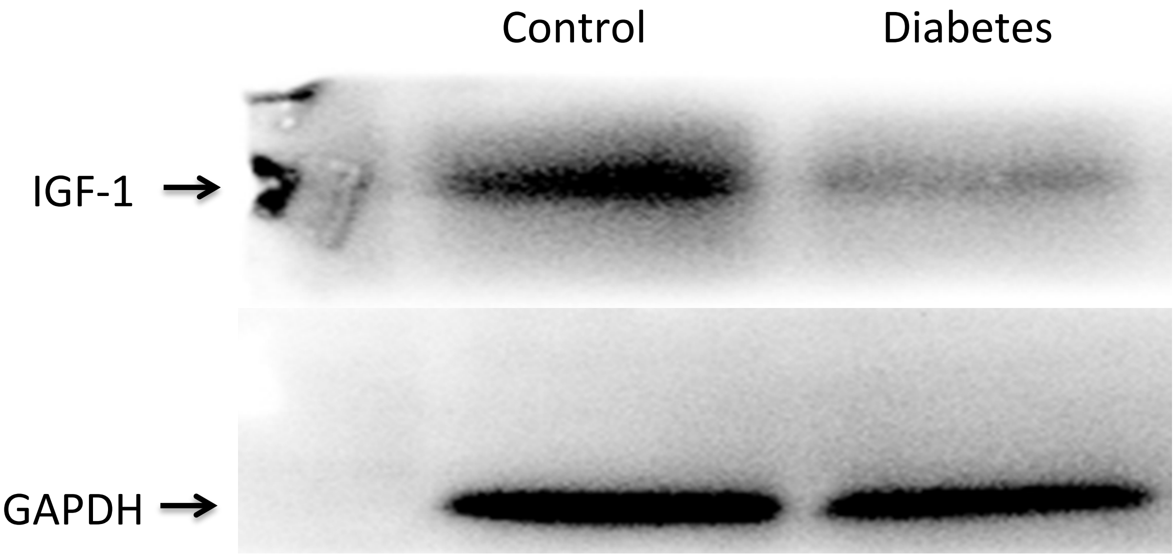

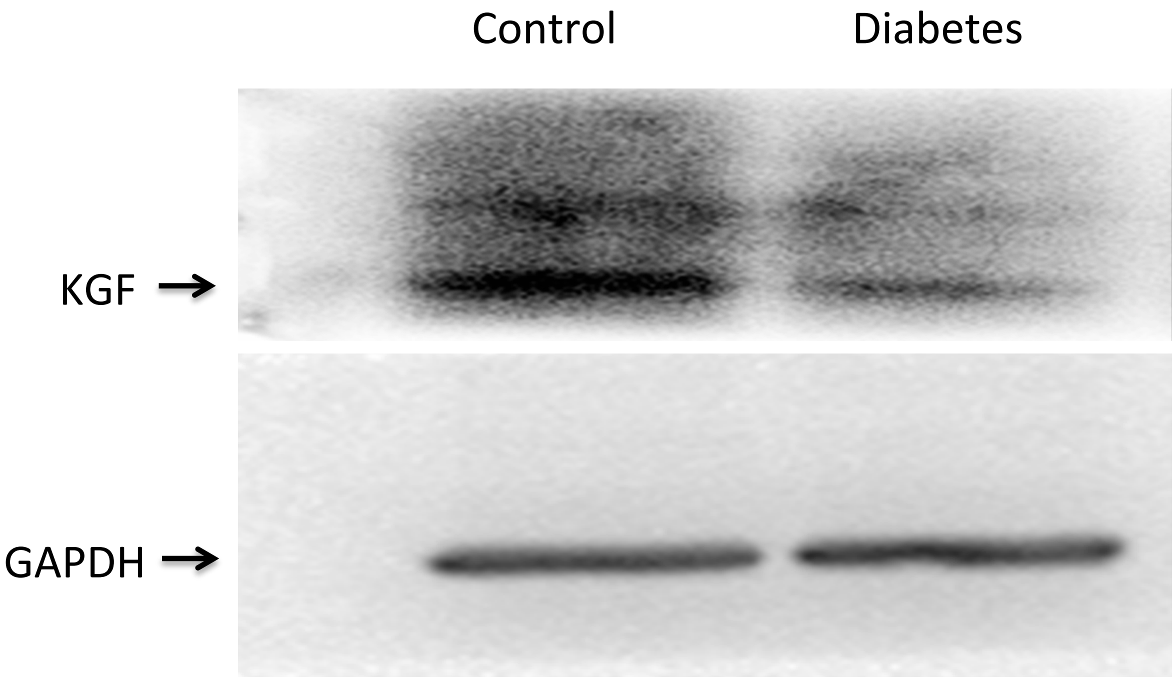

Raw data for Figure 3b

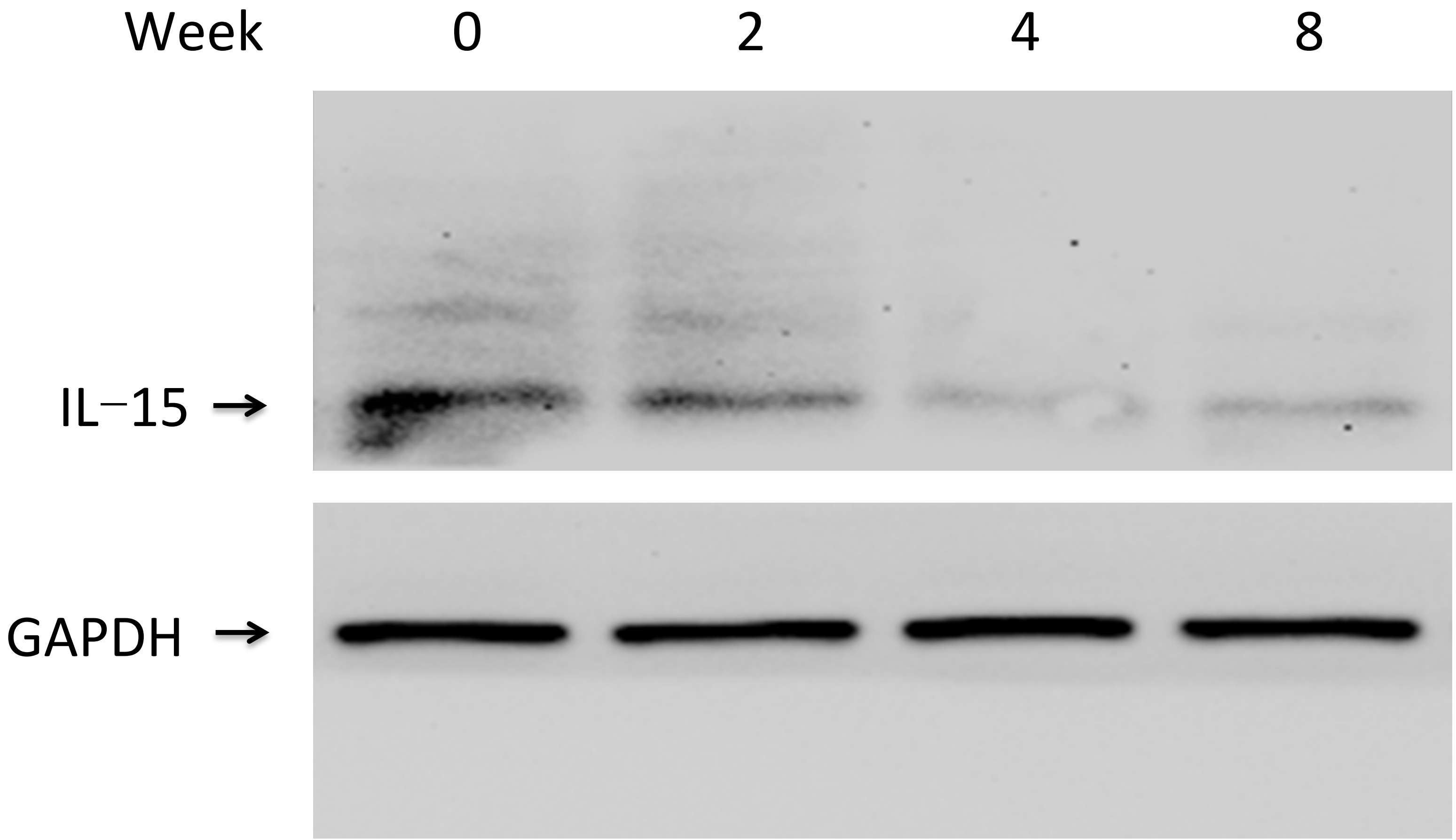

Raw data for Figure 4b

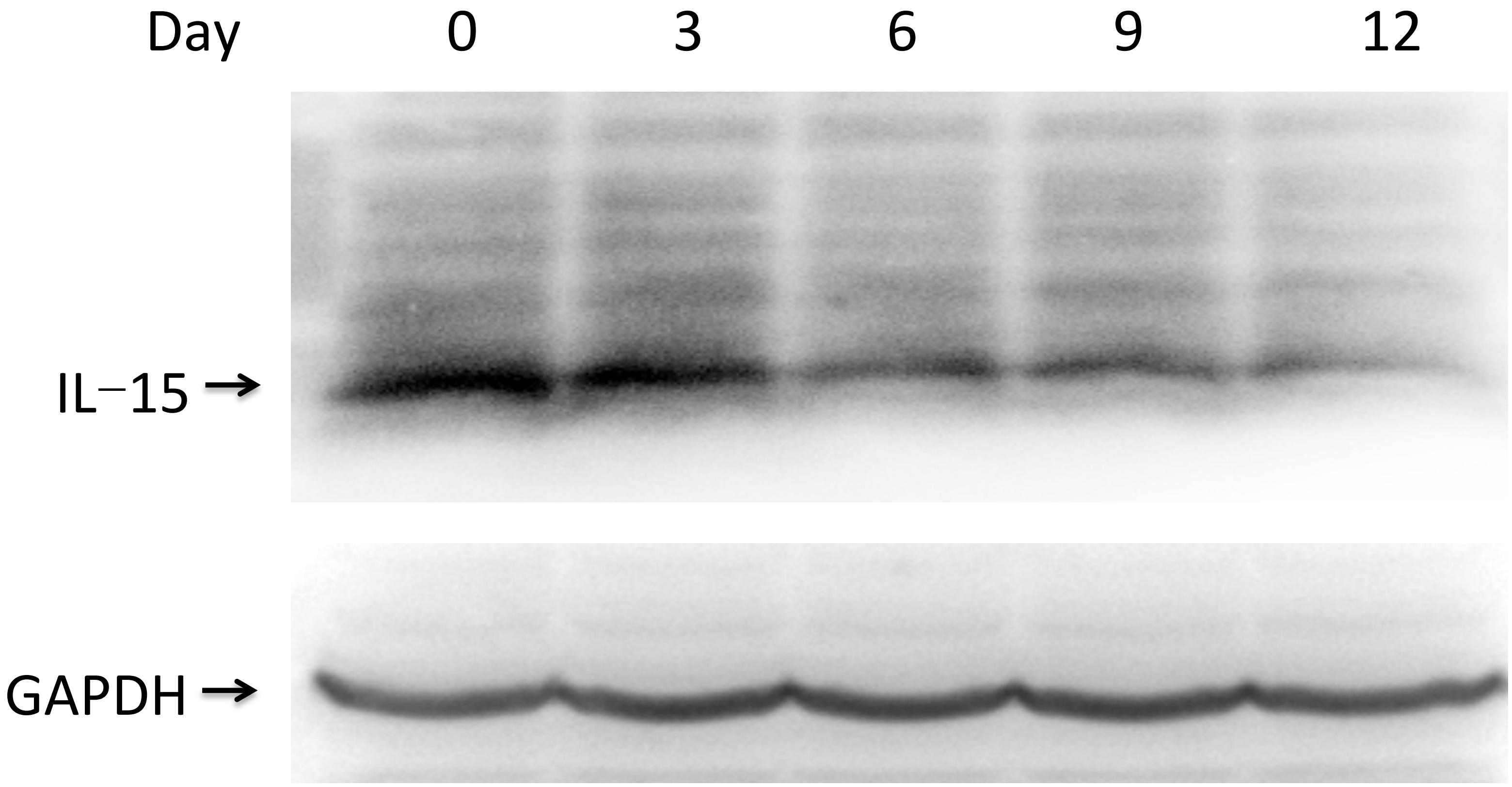

Raw data for Figure 4c

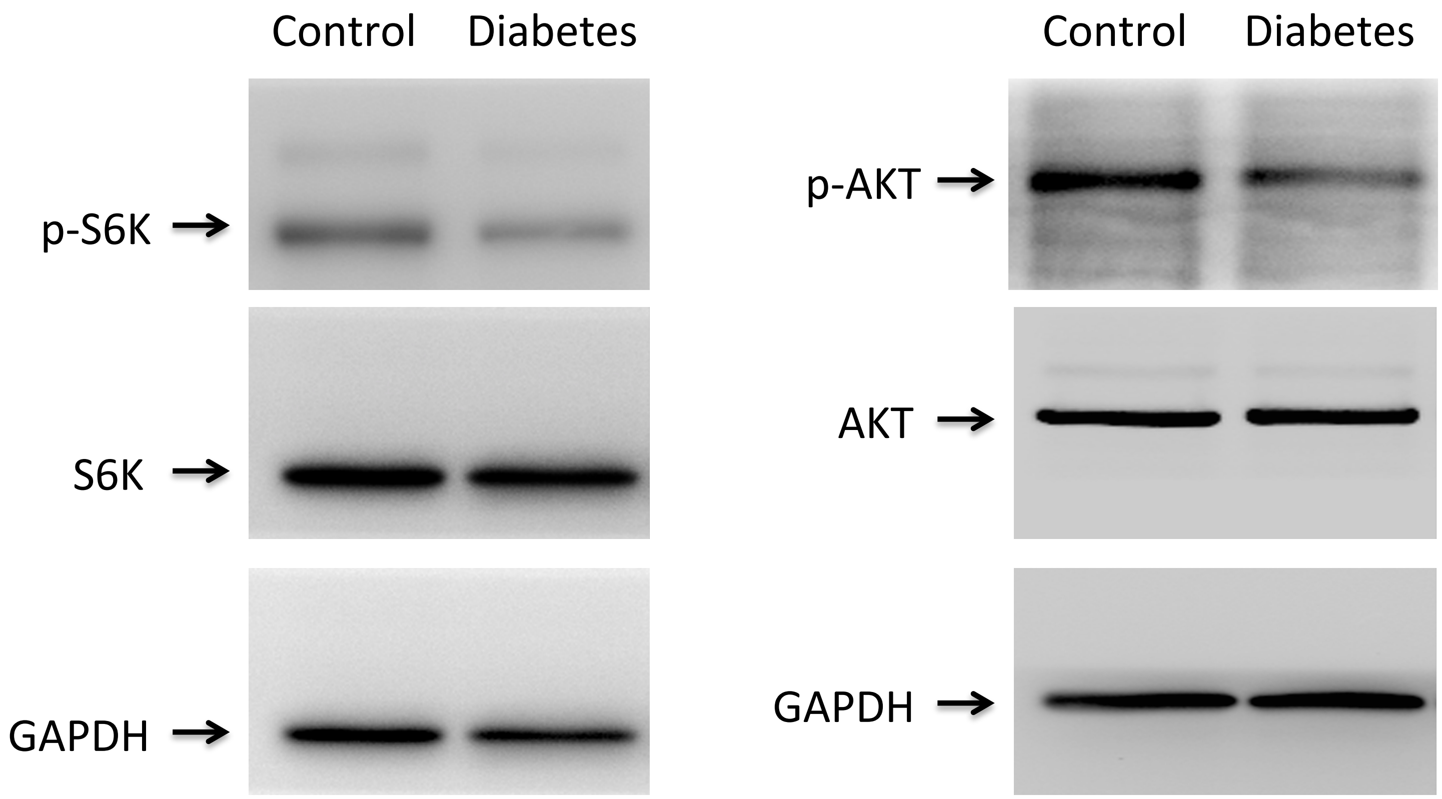

Raw data for Figure 5b

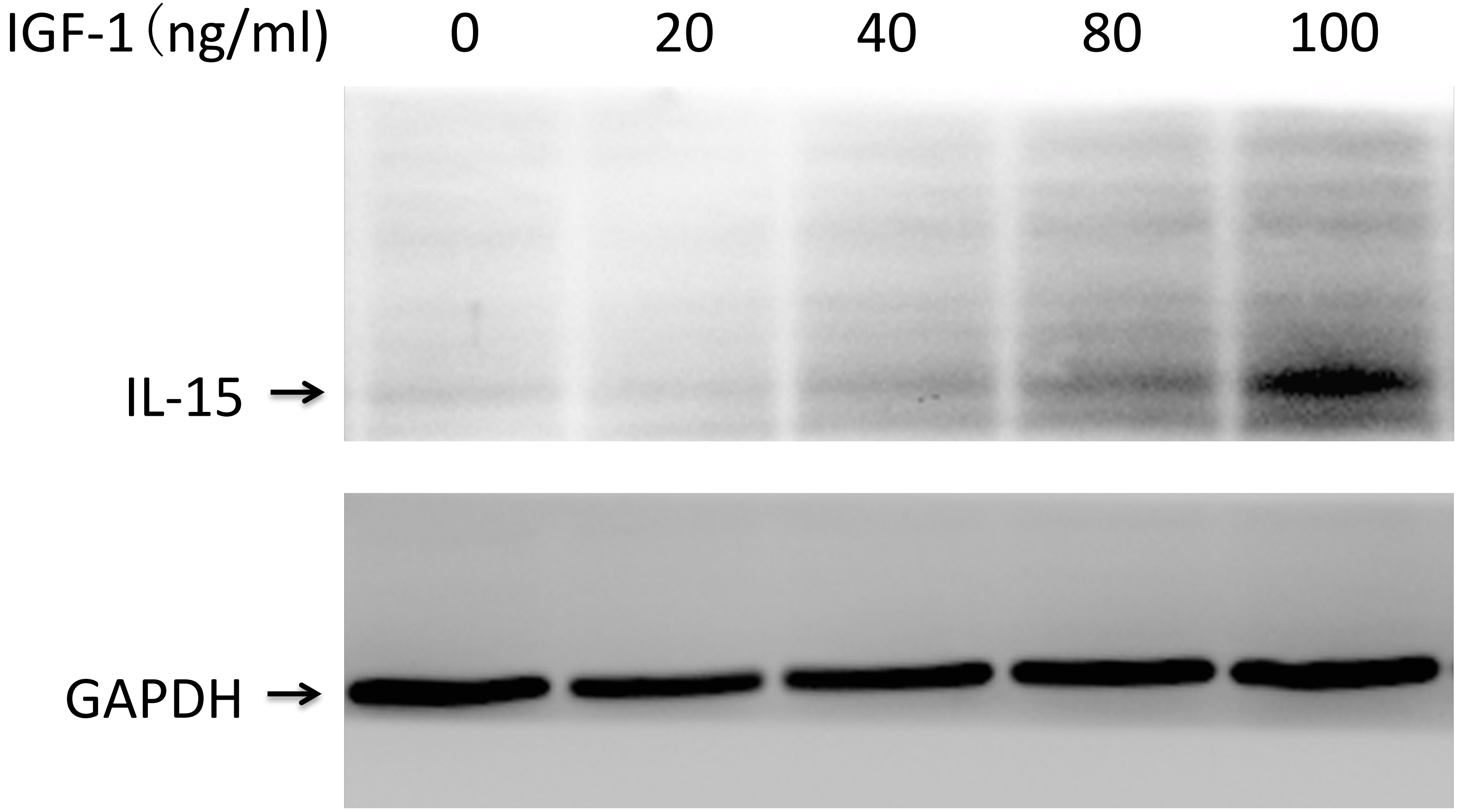

Raw data for Figure 5c

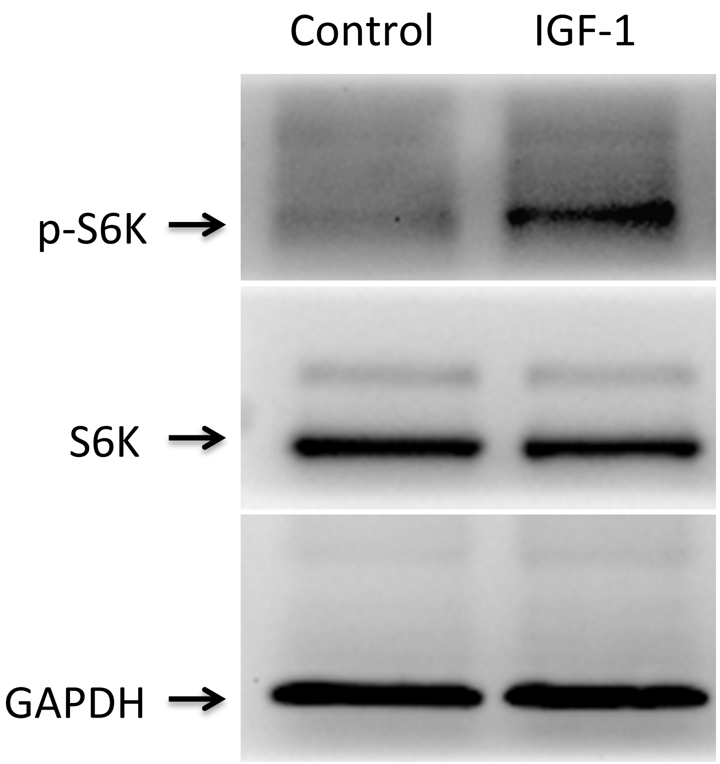

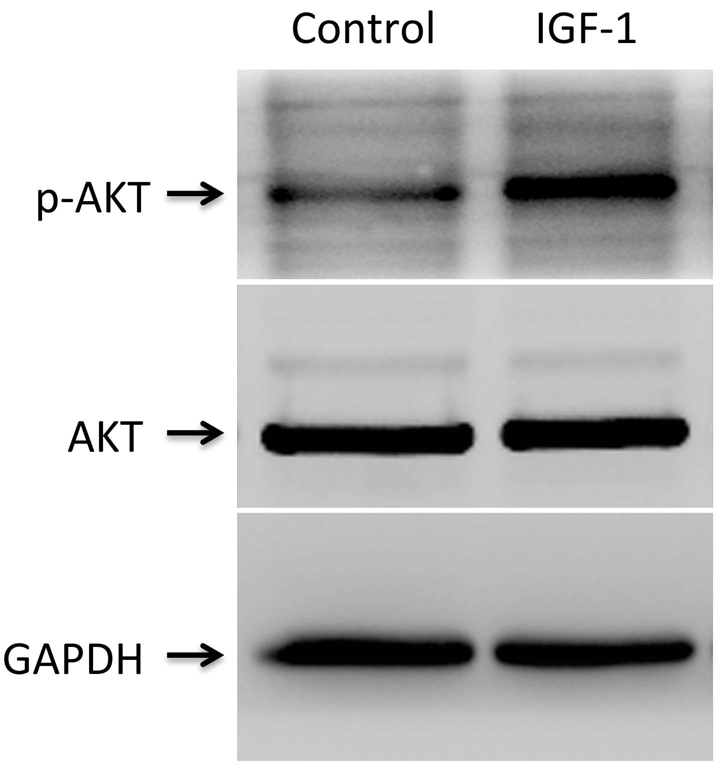

Raw data for Figure 5d

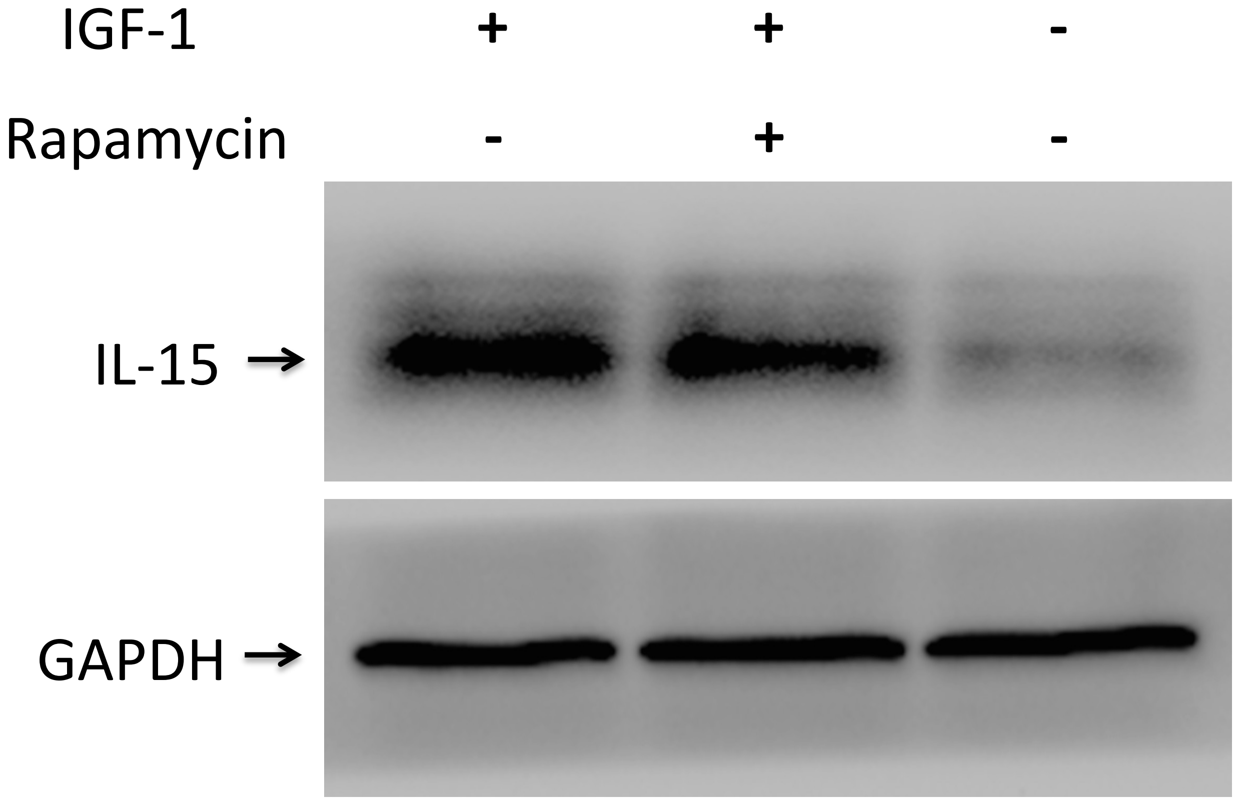

Raw data for Figure 6a

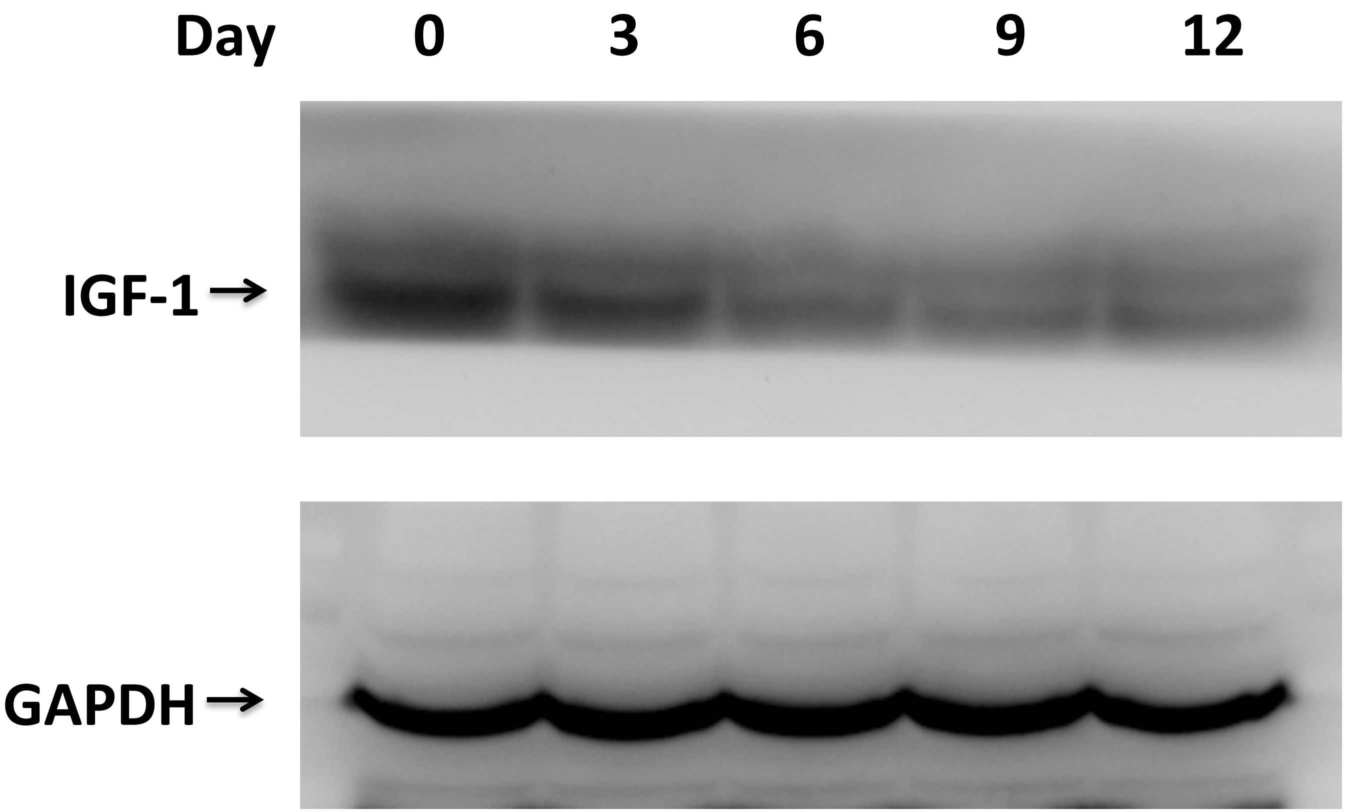

Raw data for Figure 6b

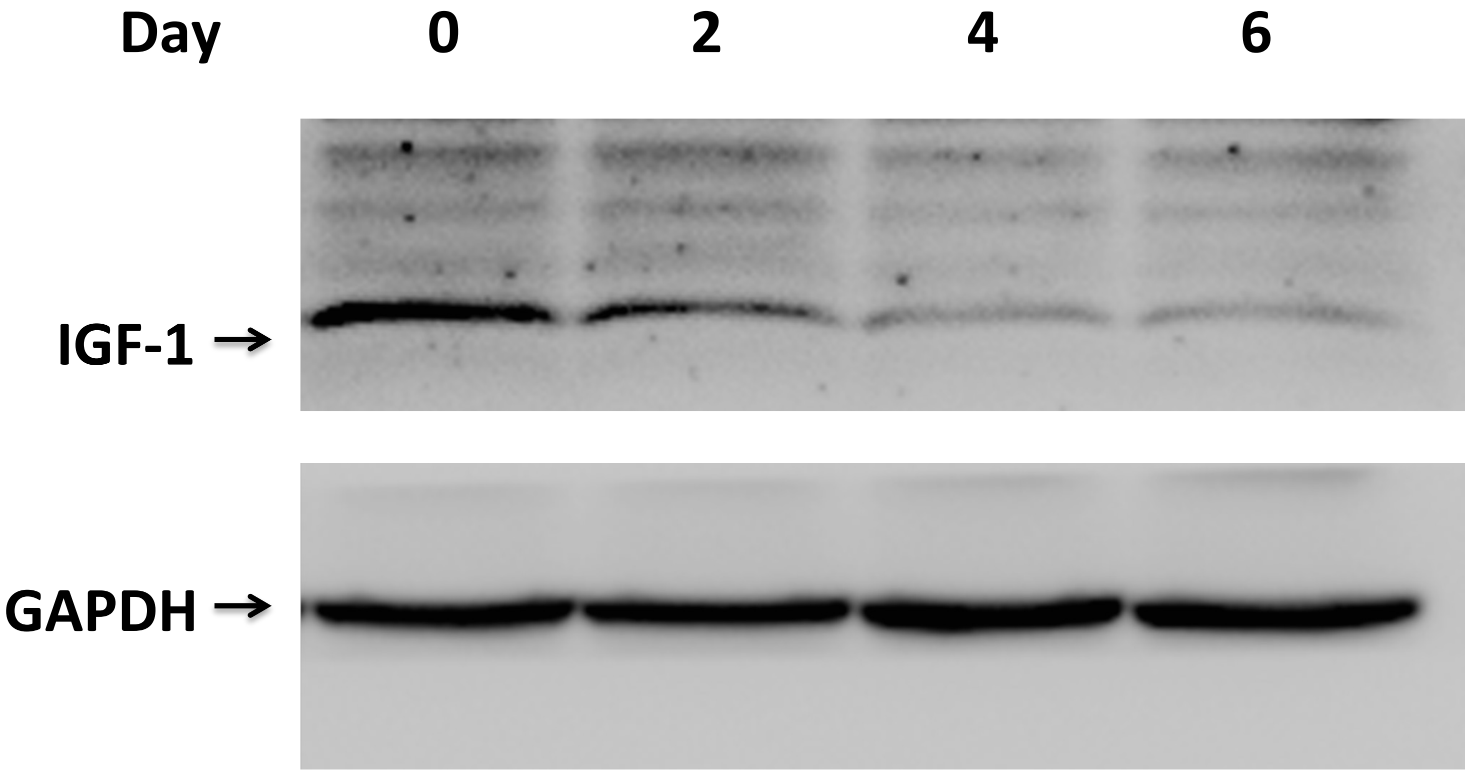

Supplement: Supplementary file 1 — Dataset 1 [file 41598_2017_5950_MOESM1_ESM.pdf]
